# Adjusting for Batch Effects in DNA Methylation Microarray Data, a Lesson Learned

**DOI:** 10.3389/fgene.2018.00083

**Published:** 2018-03-16

**Authors:** E. M. Price, Wendy P. Robinson

**Affiliations:** ^1^BC Children’s Hospital Research Institute, Vancouver, BC, Canada; ^2^Department of Medical Genetics, University of British Columbia, Vancouver, BC, Canada; ^3^Department of Obstetrics and Gynaecology, University of British Columbia, Vancouver, BC, Canada

**Keywords:** DNA methylation, 450k array, Illumina, batch correction, batch effects, ComBat, EWAS

## Abstract

It is well-known, but frequently overlooked, that low- and high-throughput molecular data may contain batch effects, i.e., systematic technical variation. Confounding of experimental batches with the variable(s) of interest is especially concerning, as a batch effect may then be interpreted as a biologically significant finding. An integral step toward reducing false discovery in molecular data analysis includes inspection for batch effects and accounting for this signal if present. In a 30-sample pilot Illumina Infinium HumanMethylation450 (450k array) experiment, we identified two sources of batch effects: row and chip. Here, we demonstrate two approaches taken to process the 450k data in which an R function, *ComBat*, was applied to adjust for the non-biological signal. In the “initial analysis,” the application of ComBat to an unbalanced study design resulted in 9,612 and 19,214 significant (FDR < 0.05) DNA methylation differences, despite none present prior to correction. Suspicious of this dramatic change, a “revised processing” included changes to our analysis as well as a greater number of samples, and successfully reduced batch effects without introducing false signal. Our work supports conclusions made by an article previously published in this journal: though the ultimate antidote to batch effects is thoughtful study design, every DNA methylation microarray analysis should inspect, assess and, if necessary, account for batch effects. The analysis experience presented here can serve as a reminder to the broader community to establish research questions *a priori*, ensure that they match with study design and encourage communication between technicians and analysts.

## Introduction

Advances in large-scale genomic technologies make it relatively easy for investigators to generate “big data” to explore a range of novel biological questions. Given the cost of such experiments and susceptibility to p-hacking (i.e., mining data until a significant result is achieved) (Nuzzo, 2014), it is important to establish appropriate study design *a priori*, including both the experimental setup as well as data analysis approach. Pilot studies give researchers such an opportunity; to determine effect size, develop processing pipelines and statistical analyses, produce preliminary data for grant applications, and ultimately assess whether an experiment warrants commitment of additional time and resources. Through a recent pilot study using a popular DNA methylation (DNAm) microarray platform, our group learned an unanticipated lesson, with implications for those who process and analyze DNAm microarray data.

The Illumina Infinium HumanMethylation450 BeadChip (450k array) (Bibikova et al., 2009) has been employed to assess DNAm in close to 1000 experimental series listed in NCBI’s GEO (the Gene Expression Omnibus, see GPL13534) (Edgar et al., 2002), making it the dominant platform for EWAS. This microarray uses oligonucleotide probes to assess the level of methylation in bisulfite-converted DNA at more than 450,000 CpG sites throughout the human genome. The platform accommodates measurement of 12 samples on a single “chip,” organized into two columns of six rows. Like other microarrays, the 450k array has been found to be subject to batch effects [i.e., technical – as opposed to biological – sources of data variation (Leek et al., 2010)] due to for example, the processing of samples on different days, use of different reagent lots or the distribution of samples across chips (Harper et al., 2013; Michels et al., 2013; Mill and Heijmans, 2013; Lehne et al., 2015). Adding batch variables into statistical models or removing batch signal prior to hypothesis testing are two of the approaches used to account for unwanted technical signal (Nygaard et al., 2016). However, several groups have cautioned that some methods used to adjust high-throughput data for batch effects can introduce false biological signal (Harper et al., 2013; Buhule et al., 2014; Nygaard et al., 2016).

In a 2014 issue of this journal, Buhule et al. (2014), describe an experience with the bioinformatic tool *ComBat* to remove chip- and row-effects from 450k data generated from the blood of lean and obese men. In the initial study, termed “sample one,” all lean samples were run on the same four 450k chips while all obese samples were run on another four 450k chips; in other words, the biological variable of interest (obese vs. lean) was completely confounded with a technical variable (chip). Before batch correction, 25,650 sites (FDR < 0.05) were identified as differentially methylated between lean and obese individuals, but this increased to 94,191 sites after batch correction. Suspicious of these results, the authors regenerated their data using a stratified randomization design that distributed obese and lean samples equally across 450k chips (“sample two”). With this balanced study design, no sites were differentially methylated between lean and obese patients before or after *ComBat* batch correction, indicating that (i) sites identified as differentially methylated in sample one were due to batch effects and (ii) applying *ComBat* to an unbalanced study design can introduce false signal.

In this Perspective article, we describe our experience with batch correction in a pilot study, which in many ways mirrors that of Buhule et al. (2014). We present the initial study design and analysis, technical issues encountered, and a revised approach that used *ComBat* to removed batch effects without introducing false signal. While the success of the revised analysis is encouraging, it is alarming that thousands of false discoveries might have been claimed if the analysis had been limited to standard processing pipelines. We aim to support the cautionary messages of others (Harper et al., 2013; Buhule et al., 2014; Nygaard et al., 2016), and implore users to explore, be skeptical and monitor every step of DNAm microarray data analysis.

## Biological Motivation for Our Pilot Study

The biological motivation for our pilot study was to clarify whether patterns of DNAm varied in association with genotype at two loci in the human genome. The genetic variants of interest were located on chromosome 1 within the gene coding for MTHFR. MTHFR catalyzes a reaction that commits methyl groups to the methylation cycle in one carbon metabolism, and two polymorphisms in *MTHFR*, 677C > T (rs1801133) (Frosst et al., 1995) and 1298A > C (rs1801131) (van der Put et al., 1998), reduce its enzymatic activity to about 45 and 68%, respectively (Weisberg et al., 2001). It has been suggested that the association of the “high-risk” homozygous alterative *MTHFR* genotypes (677TT or 1298CC) with increased disease risk [e.g., pregnancy complications (Yadav et al., 2015) and adult cardiovascular disease (Gao et al., 2014)], may be due to altered DNAm capacity (Stern et al., 2000; Friso et al., 2002; Castro et al., 2004; Narayanan et al., 2004; Shelnutt et al., 2004; Blom et al., 2006; Axume et al., 2007). To assess the association of DNAm with high-risk *MTHFR* genotypes during pregnancy, we used the 450k array to compare placental DNAm patterns (from a pool of 3 sites per placenta) between three different *MTHFR* genotype groups: variant 677 (*n* = 10; 677TT and 1298AA), variant 1298 (*n* = 10; 677CC and 1298CC), and reference (*n* = 10; 677CC and 1298AA). These 30 samples were randomly distributed within a larger batch of 84 samples run across seven 450k chips and processed following standard Illumina protocols. This design maximized cost-effectiveness by allowing several subsets of the 84 samples to be analyzed to address separate research questions.

## Initial Processing of 450k Data (*n* = 30)

**FIGURE 1:**
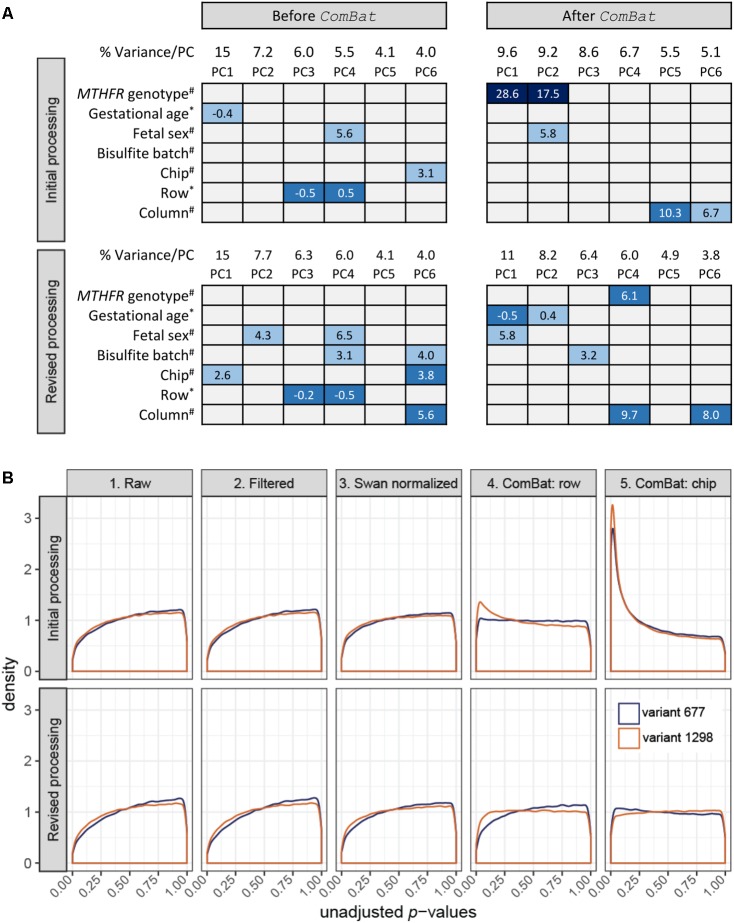
Data monitoring in the initial and revised processing approaches. **(A)** Association of top PC loadings with biological variables: (i) *MTHFR* genotype group, (ii) gestational age at birth, and (iii) fetal sex; and batch variables: (i) bisulfite conversion batch, (ii) chip, (iii) row position, and (iv) column position in the initial processing (top; *n* = 30 samples) and the revised processing (bottom; *n* = 30 samples subset from 59) before and after *ComBat* correction. Box colors indicate significance of association based on unadjusted *p*-values: dark blue *p* ≤ 0.001, mid-blue *p* ≤ 0.01, light blue *p* ≤ 0.05, gray *p* > 0.05, and contain the associated test statistic: ANOVA *F*-statistic for categorical variables (#) and Spearman’s rho for continuous/ordinal variables (∗). **(B)** At each data state, a linear model was fit to test for differential methylation between each variant *MTHFR* group vs. reference placenta. Unadjusted *p*-value distributions for each comparison were plotted for the initial processing (top; *n* = 30 samples) and the revised processing (bottom; *n* = 30 samples subset from 59). The number of sites tested in initial and revised processing, respectively, was *n* = 485,577 and *n* = 485,577 for the raw data, *n* = 442,389 and *n* = 442,378 for the filtered data, *n* = 442,389 and *n* = 442,378 for the Swan normalized data, *n* = 442,035 and *n* = 442,355 for the *ComBat* row corrected data, and *n* = 442,035 and *n* = 442,355 *ComBat* chip corrected data. **(B)** Created using ggplot2 (Wickham, 2009).

For initial processing, we extracted only data relating to the 30-sample *MTHFR* pilot to be processed in the R software environment (R Core Team, 2014). Analyses were performed on M values generated using the Bioconductor *methylumi* package (Davis et al., 2015), since this log2 ratio of array intensities has been shown to be valid for differential analysis of DNAm array data (Du et al., 2010). Testing for batch effects using PCA is a standard step in our DNAm processing pipeline. Orthogonal PCs are identified to reduce high-dimensional data into a lower number of dimensions accounting for the majority of data variation. In this study, the top six PCs were tested for association with three biological variables (fetal sex, gestational age at delivery and *MTHFR* genotype group) and four technical variables (bisulfite conversion batch, chip, row, and column) to suggest sources of data variation. Given that PC3 (*r*_s_ = -0.5, *p* = 0.005) and PC4 (*r*_s_ = 0.5, *p* = 0.005) were associated with the distribution of samples across rows (*n* = 6), while PC6 (*F* = 3.1, *p* = 0.023) was associated with the distribution of samples across chips (*n* = 7) (**Figure 1A**, top), we decided to remove these batch effects during data cleaning. Batch effect correction was attempted using *ComBat*, an empirical Bayes approach implemented in the R software environment (R Core Team, 2014), as this tool has previously been applied to DNAm data (Buhule et al., 2014; Lehne et al., 2015) and was specifically developed for small sample sizes (Johnson et al., 2007).

Our 450k analysis pipeline took the data through five major states:

(1) Raw data (*n* = 485,577 sites);
(2) Filtered data: removal of systemically poor-quality array probes (resulting in *n* = 442,389 remaining CpG sites) including:
  • probes targeting the sex chromosomes, *n* = 11,648;
  • sex chromosome cross-hybridizing probes (Price et al., 2013), *n* = 11,412;
  • polymorphic probes (Price et al., 2013), *n* = 19,957;
  • 65 rs probes;
  • probes with detection *p*-value > 0.01 or < 3 bead replicates in >20% of samples, *n* = 106;
(3) Swan normalized data: normalization to correct for differences in the dynamic range of Type I and Type II probes using *SWAN* (Maksimovic et al., 2012) (*n* = 442,389 CpG sites);
(4) *ComBat* row-corrected data: removal of probes with <2 values in a batch level (*n* = 354), followed by batch correction using *ComBat* (Leek et al., 2017) to correct for the location of samples in different chip rows while protecting *MTHFR* genotype group (*n* = 442,035 CpG sites);
(5) *ComBat* chip-corrected data: batch correction using *ComBat* (Leek et al., 2017) to correct for the distribution of samples across chips while protecting *MTHFR* genotype group (*n* = 442,035 CpG sites).

## Assessing the Processing of 450k Data

Following each of the five states outlined above, *limma* (Smyth, 2005) was used to apply a linear model to each CpG site to model DNAm as a function of *MTHFR* genotype group. Sex and gestational age at delivery were included as additive covariates in the model, as they were associated with top PCs (**Figure 1A**, top) and changes in DNAm have previously been found associated with these biological variables (Fuke et al., 2004; Chavan-Gautam et al., 2011; Novakovic et al., 2011; Mayne et al., 2017). Results were extracted for two comparisons, variant 677 vs. the reference group and variant 1298 vs. the reference group, which generated a *p*-value for every CpG site per comparison. The distribution of unadjusted *p*-values for each step was plotted to give an overall view of the data at each processing step (**Figure 1B**, top). As data was cleaned, normalized and corrected for batch effects, we expected *p-*value distributions would flatten toward uniform (i.e., equal likelihood of significant and non-significant tests) or may become skewed toward a higher number of *p-*values (i.e., right-skewed or left-peaking), if there were more differences in DNAm between genotype groups than expected by chance. The first three graphs of **Figure 1B** (top) show similar and slightly right-peaking distributions, suggestive of missing explanatory variables in the model, often batch effects. Correcting for chip row (graph 4) resulted in slightly left-peaking distributions and finally correcting for chip (graph 5) resulted in extremely left-peaking distributions, suggestive of many differences in DNAm between *MTHFR* genotype groups.

Principal component analysis was reapplied on data from state 5 to assess sources of variability in the “clean” data (**Figure 1A**, top). The application of *ComBat* removed variability due to chip and row, while a strong association with *MTHFR* genotype group appeared in PC1 (*F* = 28.6, *p* < 0.00001) and PC2 (*F* = 17.5, *p* < 0.00001) after batch correction. At a typical threshold of FDR < 0.05, no differences in DNAm were observed by *MTHFR* genotype group prior to batch correction (i.e., after SWAN normalization). After correction for chip and row, the data contained 9,612 differentially methylated CpG sites for the variant 677 comparison, and 19,214 sites for the variant 1298 comparison. Like Buhule et al. (2014), we were wary of the magnitude of change in differentially methylated CpG sites after correcting for batch and so re-examined the study design and processing of this pilot data.

## Revised Processing of 450k Data (*n* = 59)

**FIGURE 2:**
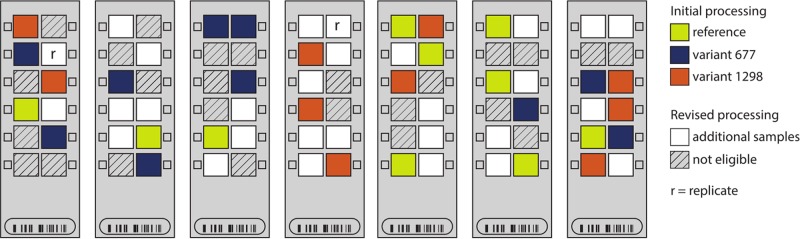
Sample distribution on seven Illumina Infinium HumanMethylation450 chips. The locations of the placental samples used in the initial processing (*n* = 30 samples) are indicated by color: yellow – reference; blue – variant 677; and orange – variant 1298. In the revised processing, all other placental samples (white) were genotyped for *MTHFR* c.677 (rs1801133) and c.1298 (rs1801131) and added to the processing (*n* = 59 samples). Non-placental and poor-quality samples were ineligible for inclusion in the revised processing (hashed).

*ComBat* requires two inputs to correct for batch effects: (i) a model describing the parameter(s) that should be protected from correction (in this case, *MTHFR* genotype group); and (ii) the batch variable to be corrected for (in this case, row and then chip). Because of the randomization of our small number of samples of interest (*n* = 30) within the larger group of samples run at the same time (*n* = 84), the distribution of any given *MTHFR* genotype group across chips and rows was sparse (**Figure 2**), leading to partial confounding of biological and technical variables. Nygaard et al. (2016) showed that the use of *ComBat* in high-dimensional datasets where the batch variable and protected variable are confounded can lead to inflation of *p-*values, and that the magnitude of the effect is related to the severity of confounding. Thus a “revised processing” was conducted including two changes to our analysis aimed at improving batch correction:

(i) Increased sample size: we made use of 29 other placental samples from within the 84-sample batch (white arrays in **Figure 2**), to increase the pre-processing sample size from 30 to 59, with a better distribution of samples across chips and rows. This also allowed for the inclusion of a technical replicate to better monitor data processing (replicate pair indicated by r’s in **Figure 2**).
  Unlike the original 30 samples, none of these additional samples was homozygous for both *MTHFR* variants and also from a healthy pregnancy. Thus, the protected parameters in the revised analysis were the sample genotypes at rs1801133 (677) and rs1801131 (1298), so that heterozygous samples could be included. This also meant that the exact biological variable of interest (*MTHFR* group) was no longer protected by *ComBat.*
(ii) Reduced sample subdivision: in running of the 450k arrays, chips stand vertically for approximately 3 h while a series of washes are applied. We hypothesized that this step may account for some of the row effect. Thus, samples were grouped into high (rows 5 and 6), mid (rows 3 and 4), and low (rows 1 and 2) locations to reduce the number of row categories that needed to be estimated (i.e., three instead of six), and thereby reduce the confounding of batch and biological variables.

During the revised processing, the number of sites went from 485,577 to 442,378 to 442,355 in the raw to filtered to combat datasets respectively. The 30 *MTHFR* samples were selected out of the larger group of 59 samples after each processing step and a linear model was fit (**Figure 1B**, bottom), as described for the initial processing. While graphs 1 through 3 mirror those of the initial processing, the distributions in graph 5 are close to uniform, suggesting that the applied models fit and that batch effects were removed. In support, the pairwise correlation of all probes in the technical replicate included in the revised processing improved slightly, from *r* = 0.99616 in the raw data to *r* = 0.99668, after batch correction. PCA confirmed the removal of row and chip effects, without introduction of a strong *MTHFR* genotype group signal (**Figure 1A**, bottom). The corrected data contained strong gestational age (PC1 *r*_s_= -0.5, *p* = 0.006; PC2 *r*_s_= 0.4, *p* = 0.042) and fetal sex (PC1 *F* = 5.8, *p* = 0.022) signal, as well as some signal associated with the technical variables bisulfite conversion batch (PC3 *F* = 3.2, *p* = 0.041) and column (PC4 *F* = 9.7, *p* = 0.004; PC6 *F* = 8.0, *p* = 0.008). This final PCA suggests that bisulfite batch and column may be additional sources of batch effects to consider in the design of DNAm microarray experiments.

## Discussion and Conclusion

When batches of experiments, such as processing date, operator, or run plate, are confounded with the variable of interest, differences between biological groups may be identified that are, in fact, artifacts (Harper et al., 2013; Buhule et al., 2014). This issue has been discussed for some time in gene expression microarray studies; a striking example was highlighted by Akey et al. (2007), who attempted to reanalyze a publicly-available dataset comparing gene expression between two ethnic groups (Spielman et al., 2007). Akey and colleagues found that most of the data for European participants was produced 2 years prior to that for Asian participants. The reanalyzed data showed that the near-complete confounding of measurement year with ethnicity was likely the source of >4,000 “differentially expressed” genes identified in the original study (Akey et al., 2007).

Another 2007 publication identified batch effects as one of the top three sources of data variability in eight of nine gene expression microarray studies examined (Leek et al., 2010). It was suggested that most, if not all, high-throughput datasets contain batch effects, and that in many cases this unwanted signal is the primary source of data variation (Leek et al., 2010). In 2006, a consortium of scientists and organizations, ran the MicroArray Quality Control or MAQC project to systematically test for batch effects in gene expression microarray data (Shi et al., 2006). From this project, guidelines and methodologies for standardized processing, reporting, and analysis of gene expression microarray data were established. *ComBat*, the R function employed in our pilot, is one such tool developed to aid researchers in correcting batch effects in ‘omics’ datasets.

But analysts, reviewers and readers should be wary that even by employing tools like *ComBat*, it may not be possible to remove technical signal when batches are confounded with variables of interest. Our experience adds to the growing set of empirical and simulated examples demonstrating that the application of *ComBat* to high-throughput data with uneven study design may, in fact, introduce false signal. Interestingly, if, as suggested by Nygaard et al. (2016), we had used row and chip as additional covariates in our linear model instead of adjusting for batch effects in the initial processing, the inflation of *p-*values would have been avoided. We hope our experience will aid 450k users to better design their experiments and analyses, especially in cases of limited samples size due to rare exposures/phenotypes, difficult to access tissues (e.g., brain, liver, fetal tissues) or budget constraints. For critical evaluation of EWAS, manuscripts must include details of study design along with the approach to mitigate batch effects, and metrics (e.g., replicates, PCA, *p-*value distributions) used to assess data processing. Furthermore, identified significant differences should ideally be verified using a different assay as well as validated in a distinct cohort (Michels et al., 2013).

Many journals and funding agencies now require data to be posted to public repositories, a key resource in which to test replication in populations with similar and different characteristics (Munafò et al., 2017). However, a systematic review (Piwowar, 2011) suggested that though data sharing is on the rise, only 45% of gene expression microarray datasets were deposited in NCBI’s GEO (Edgar et al., 2002) or EBI’s ArrayExpress (Parkinson et al., 2006). Furthermore, the degree of compliance with data sharing is variable; for example, of nearly 2,500 450k samples in GEO in 2014, close to 1,000 did not report sex on a per-individual basis (Cotton et al., 2015). For shared data to be used to its full potential, a truly altruistic approach is needed: accept and publish negative findings, describe challenges, detail the processing pipeline and report demographics (tissue, sex, age, ethnicity etc.) as well as technical features (design, batches, processing steps etc.) on a per-sample level.

## Materials

Raw and processed data for the samples used in this study were deposited in NCBI’s Gene Expression Omnibus (Edgar et al., 2002) and are accessible through GEO Series accession number GSE108567^1^

## Ethics Statement

This study was carried out in accordance with the recommendations of the University of British Columbia/Children’s Hospital and Women’s Health Centre of British Columbia Research Ethics Board (certificate: H04-70488) with written informed consent from all subjects. All subjects gave written informed consent in accordance with the Declaration of Helsinki. A subset of cases were de-identified biospecimens obtained from the BC Children’s and Women’s Embryo and Fetal pathology lab and unlinked to clinical data. The protocol was approved by the University of British Columbia/Children’s Hospital and Women’s Health Centre of British Columbia Research Ethics Board.

## Author Contributions

EP conceived the study, analyzed and interpreted the data and drafted the manuscript. WR conceived the study and contributed to data interpretation. All authors read and approved the final manuscript.

## Conflict of Interest Statement

The authors declare that the research was conducted in the absence of any commercial or financial relationships that could be construed as a potential conflict of interest.

## Footnotes

1 http://www.ncbi.nlm.nih.gov/geo/query/acc.cgi?acc=GSE108567

## Abbreviations

DNAm: DNA methylation
EWAS: epigenome-wide association study
FDR: false discovery rate
GEO: gene expression omnibus
MTHFR: 5,10-methylenetetrahydrofolate reductase
PC: principal component
PCA: principal component analysis
